# Blocking CCL5-CXCL4 heteromerization preserves heart function after myocardial infarction by attenuating leukocyte recruitment and NETosis

**DOI:** 10.1038/s41598-018-29026-0

**Published:** 2018-07-13

**Authors:** Tanja Vajen, Rory R. Koenen, Isabella Werner, Mareike Staudt, Delia Projahn, Adelina Curaj, Tolga Taha Sönmez, Sakine Simsekyilmaz, David Schumacher, Julia Möllmann, Tilman M. Hackeng, Philipp von Hundelshausen, Christian Weber, Elisa A. Liehn

**Affiliations:** 1 Cardiovascular Research Institute Maastricht (CARIM), Department of Biochemistry, Maastricht University, Maastricht, The Netherlands; 2 Institute for Cardiovascular Prevention (IPEK), LMU Munich, Munich, Germany; 3 Institute for Molecular Cardiovascular Research (IMCAR), RWTH Aachen University, Aachen, Germany; 4 Department of Experimental Molecular Imaging, RWTH Aachen University, Aachen, Germany; 5 Victor Babes National Institute of Pathology, Bucharest, Romania; 6 Department of Oral and Maxillofacial Surgery, Karlsruhe City Hospital of Freiburg University, Freiburg, Germany; 7 Department of Oral and Maxillofacial Surgery, University Medical Center Hamburg-Eppendorf, Hamburg, Germany; 8 DZHK (German Centre for Cardiovascular Research), partner site Munich Heart Alliance, Munich, Germany; 9 Department of Cardiology, Pulmonology, Angiology and Intensive Care, University Hospital Aachen, Aachen, Germany; 10 Human Genetic Laboratory, University of Medicine and Pharmacy, Craiova, Romania

## Abstract

Myocardial infarction (MI) is a major cause of death in Western countries and finding new strategies for its prevention and treatment is thus of high priority. In a previous study, we have demonstrated a pathophysiologic relevance for the heterophilic interaction of CCL5 and CXCL4 in the progression of atherosclerosis. A specifically designed compound (MKEY) to block this CCL5-CXCR4 interaction is investigated as a potential therapeutic in a model of myocardial ischemia/reperfusion (I/R) damage. 8 week-old male C57BL/6 mice were intravenously treated with MKEY or scrambled control (sMKEY) from 1 day before, until up to 7 days after I/R. By using echocardiography and intraventricular pressure measurements, MKEY treatment resulted in a significant decrease in infarction size and preserved heart function as compared to sMKEY-treated animals. Moreover, MKEY treatment significantly reduced the inflammatory reaction following I/R, as revealed by specific staining for neutrophils and monocyte/macrophages. Interestingly, MKEY treatment led to a significant reduction of citrullinated histone 3 in the infarcted tissue, showing that MKEY can prevent neutrophil extracellular trap formation *in vivo*. Disrupting chemokine heterodimers during myocardial I/R might have clinical benefits, preserving the therapeutic benefit of blocking specific chemokines, and in addition, reducing the inflammatory side effects maintaining normal immune defence.

## Introduction

Myocardial infarction (MI) is among the most common causes of death in developed countries. The acute lack of blood supply following the blockage of the atherosclerotic coronary vessels induces the death of cardiomyocytes and causes a dramatic up-regulation of many inflammatory factors. Effective treatment of acute MI by reperfusion therapy promotes the restoration of blood flow to the ischemic myocardium. However, the ensuing inflammatory reactions continue to damage the heart tissue during reperfusion, which may lead to further irreversible cardiomyocyte death. On the other hand, the inflammatory reaction also serves to prepare the myocardial tissue and its resident cells for repair^1^. For example, neutrophils were recently shown to polarize macrophages towards a reparative phenotype^2^, indicating an ambivalent role of neutrophil in MI-related inflammation. Eventually, reparative mechanisms lead to the replacement of the injured area with scar tissue^3–5^.

Despite the discovery of the cardioprotective effects of the innate adaptive responses evoked by different conditioning strategies (pre-, post- and remote conditioning) and intense research to identify key cellular mechanisms and drug targets^6,7^, the currently available treatment for myocardial ischemia/reperfusion (I/R) injury is still suboptimal to modulate the phases and actors of post-MI inflammation in order to sufficiently decrease subsequent scar size^3^. This warrants a continued quest for new therapeutic cardioprotective strategies to control the inflammatory reaction of myocardial I/R injury.

Chemokines are small cytokines that bind to specific G protein-coupled receptors (GPCR) and exert distinct functions, e.g. regulating the activation of leukocytes and coordinating their trafficking to the sites of inflammation^8^. The benefits of the antagonism of the chemokine system have been demonstrated in various studies employing animal models of myocardial infarction^3,9–13^. However, direct inhibition or genetic deficiency of chemokines or their receptors may also be accompanied with immunologic side effects, e.g. impaired immune defense against viral and bacterial pathogens or increased autoimmune organ injury^14–17^. The chemokines CCL5 (RANTES) and CXCL4 (platelet factor 4) are stored in the secretory α-granules of platelets and were shown to be potent neutrophil and monocyte attractants^10,18^. In addition, these chemokines were shown to undergo heterophilic interactions and the CCL5/CXCL4 heteromers are particularly potent in recruiting monocytes and neutrophils^19–22^. Besides leukocyte recruitment, the CCL5/CXCL4 complex was also identified to induce the release of neutrophil extracellular traps (NETs) from neutrophils, upon physical contact with platelets^23^. A cyclic peptide, termed MKEY that specifically blocked the heterophilic interaction between CCL5 and CXCL4 could prevent the enhancement of CCL5-induced leukocyte recruitment by CXCL4^20,21^ as well as the release of NETs. Moreover, administration of MKEY in mice attenuated vascular remodeling processes e.g. atherosclerosis and aortic aneurysm and LPS- and ventilator-induced lung injury^20,21,23,24^. Of note, the disruption of CCL5/CXCL4 heteromerization did not interfere with systemic immune responses, nor with T cell proliferation or clearance of viral infection^20^. In addition, the MKEY peptide did not have any effects in mice lacking either CCL5 or CXCL4.

Since experimental inhibition of CCL5 showed cardioprotective effects during myocardial reperfusion^12,25,26^ and the formation of NETs is also associated with its pathophysiology^27^, we hypothesize that blocking CCL5-CXCL4 interactions will have therapeutic benefits in the early phase of MI, with potential impact on injury progression and healing after I/R injury. In this study, we evaluated the effects of MKEY treatment on myocardial tissue and cardiac function in a mouse model of myocardial I/R.

## Results

### Effects of MKEY on cardiomyocyte survival and early infarction area

**Figure 1:**
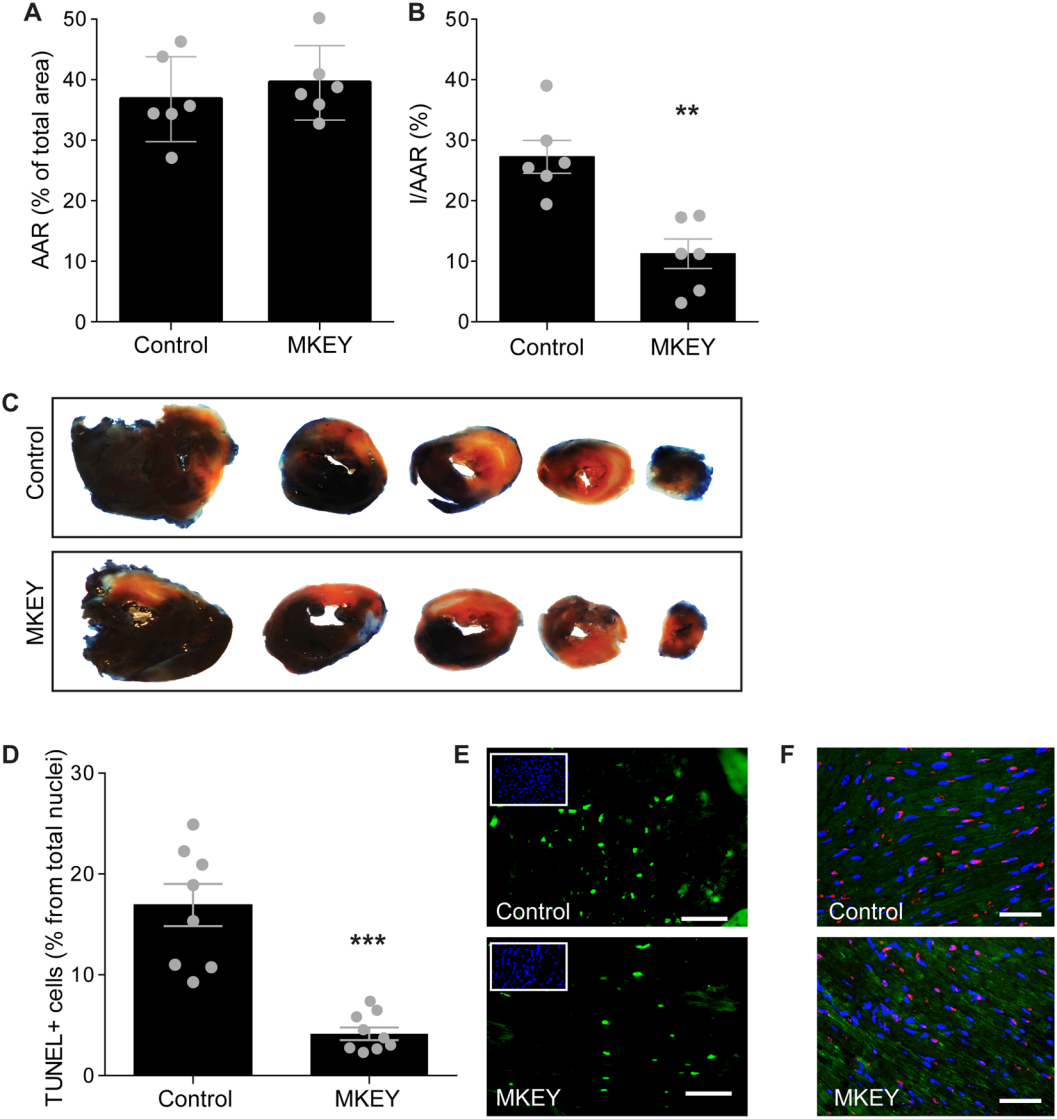
Analysis of infarcted area after MKEY treatment. Area at risk (AAR) as % total area (**A**) and infarcted area as % of AAR (**B**) were determined by Evans-blue perfusion and tetrazolium staining 1d after injury in control and MKEY-treated mice. (**C**) Representative heart sections from both groups are shown. *p < 0.05 vs. control (n = 6); (**D**) Quantification of the number of apoptotic cells as determined by TUNEL staining in MKEY- and control-treated mice. *p < 0.05 vs. control (n = 8); (**E**,**F**) Representative images of TUNEL staining of infarct tissue. Scale bar 50 µm. (**E**) TUNEL-positive cells are green, inset: control staining. (**F**) TUNEL-positive cells are red and cardiomyocytes are visualised using α-actinin (green). Nuclei were stained using DAPI (blue).

In a previous report, we performed pharmacokinetic studies, showing that the plasma half-life of MKEY is 3.02 hours, enough to ensure effective plasma concentrations of peptide inhibitor for at least 12 hours. For long-term experiments (up to 7 days), continuous intravenous infusion was accomplished using osmotic minipumps connected to a jugular vein catheter, while intraperitoneal injections were used for short-term experiments (Suppl. Fig. 1). One day after reperfusion, the effect of MKEY on cardiomyocyte survival was determined by tetrazolium staining. Evans-blue perfusion showed no differences in area at risk (AAR) between the mice treated with MKEY or control sMKEY (MKEY: 39.5 ± 2.5% vs. control: 36.8 ± 2.9%, n = 6, Fig. 1A). Tetrazolium staining showed a significant preservation of viable area in MKEY treated group, compared with control (MKEY: 11.2 ± 2.4% vs. control: 27.3 ± 2.7%, p ≤ 0.01, n = 6, Fig. 1B,C). Staining of apoptotic cells (TUNEL) confirmed these results, since the number of apoptotic cells were significantly lower in MKEY, compared with control treated mice (MKEY: 4.2 ± 0.6% n = 8, vs. control: 16.9 ± 2.1, p ≤ 0.001, n = 9, Fig. 1D–F). These cells were identified as cardiomyocytes by double staining with α-actinin and TUNEL.

### Effects of MKEY treatment on heart function after I/R

**Figure 2:**
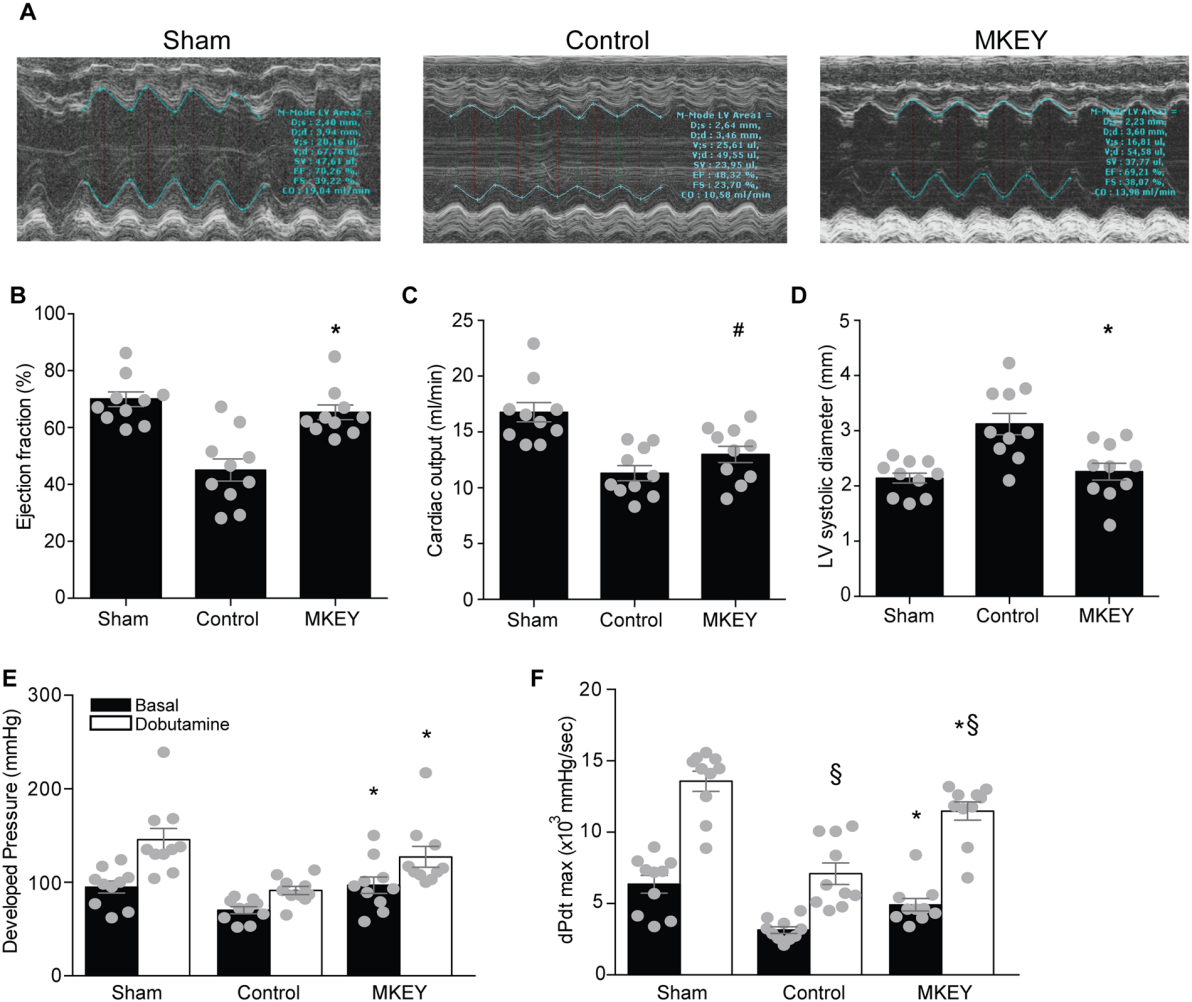
Effects of MKEY treatment on heart function after I/R. Representative images of echocardiography (**A**) and quantification of ejection fraction (**B**) cardiac output (**C**) and preserved systolic (**D**) ventricular dimensions in MKEY-treated, compared to sMKEY-treated control mice, one week after I/R. Left ventricular developed pressure (**E**) and contraction (**F**) as well as the response to dobutamine stimulation (white bars **E**,**F**), were assessed using intraventricular Millar catheter. ^#^p < 0.05 vs. sham; *p < 0.05 vs. control; ^§^p < 0.05 vs. unstimulated (n = 10).

To examine the effects of MKEY in healing following I/R, 8 week-old male mice were treated with MKEY or sMKEY to block specific CCL5-CXCR4 interactions. Functional parameters of the heart were evaluated by echocardiography after myocardial I/R or sham operation (Fig. 1A and Suppl. Videos 1–3). Before MI, no significant differences were observed among the groups. After I/R, MKEY treatment preserved the ejection fraction, as compared to sMKEY control mice (Table 1 and Fig. 2B). Analysis of cardiac output showed compensation in all groups, at similar heart rates (Table 1 and Fig. 2C). Moreover, despite unchanged heart weight (Table 1), the hearts treated with MKEY did not dilate after ventricular remodelling compared with control hearts, as measured by left ventricular diameter in systole (Table 1 and Fig. 2D) and diastole (Table 1).

**Table 1:** Echocardiography parameters.

	Sham (n = 10)	Control (n = 10)	MKEY (n = 10)	P value (ANOVA)
Ejection Fraction (%)	69.9 ± 2.56	45.1 ± 3.86	65.4 ± 2.59	<0.0001
Cardiac Output (ml/min)	16.7 ± 0.86	11.3 ± 2.13	12.9 ± 2.29	<0.0001
LVEDD (mm)	3.66 ± 0.09	4.09 ± 0.17	3.47 ± 0.57	0.0189
LVESD (mm)	2.14 ± 0.09	3.12 ± 0.19	2.26 ± 0.15	0.0001
Heart rate (bpm)	409 ± 11	382 ± 16	386 ± 12	ns
Heart weight (mg)	120 ± 7	113 ± 7	112 ± 5	ns

LVEDD: left ventricular end-diastolic diameter; LVESD: left ventricular end-systolic diameter; ns: no significance.

Similar results were obtained by invasive intraventricular measurements using small-animal pressure transducer catheters, one week after myocardial I/R. Treatment with MKEY preserved the left ventricular developed pressure both in the absence or presence of dobutamine (Table 2 and Fig. 2E), whereas heart rates did not differ among groups (Table 2). The maximal (dP/dt_max_) and minimal (dP/dt_min_) pressure change in left ventricle measurements confirmed these results. Moreover, treatment with MKEY preserved contraction (dP/dt_max_, Table 2 and Fig. 2F) as well as the response to dobutamine stimulation (white bars, Table 2 and Fig. 2F). The compounds had no effect on the differential blood pressure, which did not differ between the groups with or without dobutamine treatment (Suppl. Fig. 2).

**Table 2:** Intraventricular pressure measurements.

	Sham (n = 10)		Control (n = 10)		MKEY (n = 10)		P value (*control)
	Basal	Dobutamine	Basal	Dobutamine	Basal	Dobutamine	
Developed Pressure (mmHg)	94.9 ± 6.38	145.5 ± 12.21	70.1 ± 3.68	91.3 ± 4.32	96.9 ± 8.65*	127 ± 11.1	<0.05
dPdt max (mmHg/s)	6357 ± 624	13569 ± 713	3138 ± 232	7093 ± 755	4911 ± 440*	11480 ± 644	<0.05
dPdt min (mmHg/s)	−4595 ± 667	−7795 ± 461	−2850 ± 181	−5121 ± 616	−4302 ± 425*	−6611 ± 285	<0.05
Heart rate (bpm)	235 ± 17	414 ± 30	223 ± 11	387 ± 19	221 ± 10	425 ± 22	ns

dP/dtmax: the increase in left ventricle pressure change as measure of contraction; dP/dtmin: the decrease in left ventricle pressure change as measure of relaxation; ns: no significance.

### Analysis of remodelling and the inflammatory reaction after MKEY treatment

**Figure 3:**
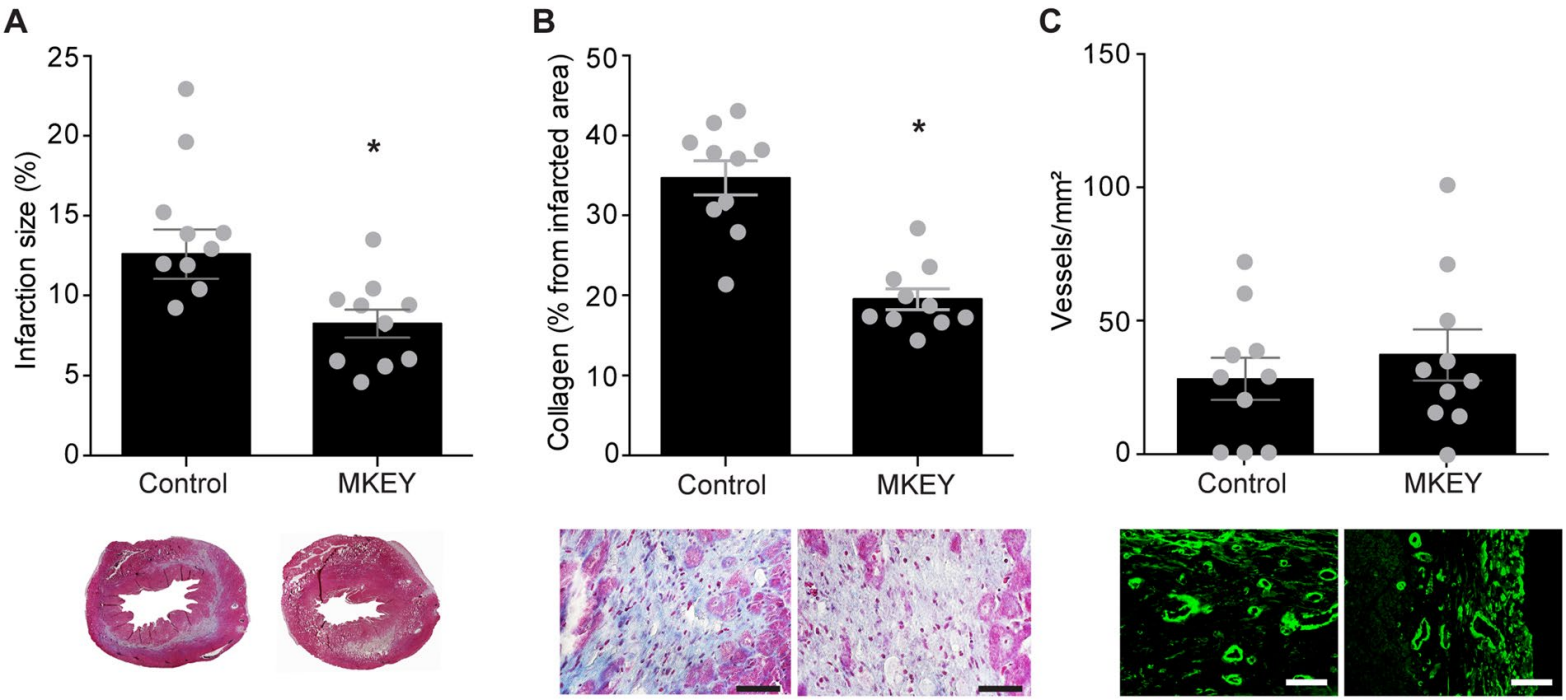
Analysis of remodeling after MKEY treatment. Infarction size (**A**) collagen deposition (**B**) and CD31^+^ collateral vessel formation (**C**) in MKEY- and control sMKEY-treated mice one week after I/R. *p < 0.05 vs. control (n = 10); Scale bar 50 µm.

**Figure 4:**
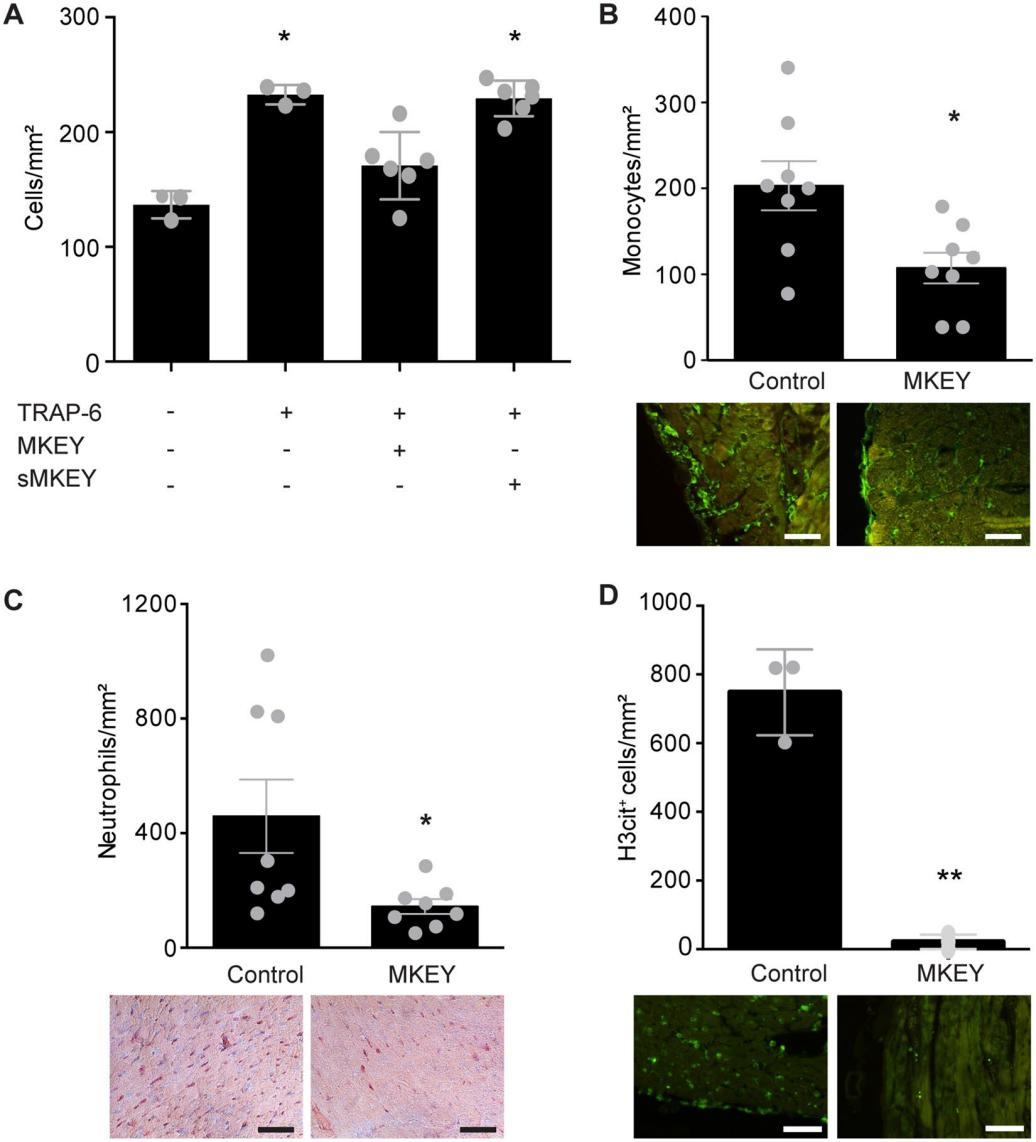
Analysis of inflammatory reaction after MKEY treatment. Human neutrophil recruitment to human platelets immobilized on collagen-coated glass slides under flow conditions (1 dyne/cm^2^), without or with TRAP-6 activation (50 µM) in presense of sMKEY (control) or MKEY (**A**) p = 0.001 (n = 3–6), monocyte/macrophage infiltration ((**B**) F4/80 staining), neutrophil infiltration ((**C**) specific esterase staining), and NET formation ((**D**) H3cit staining), in MKEY- and sMKEY-treated mice, one day after I/R; *p < 0.05 vs. control (n = 8); Scale bar 50 µm.

In accordance with the functional data one week after I/R, mice treated with MKEY showed a significant decrease in the size of the infarcted area as compared to control peptide sMKEY-treated animals (8.2 ± 0.9% vs. 12.6 ± 1.5%, respectively, p < 0.05, n = 10, Fig. 3A), with significantly reduced collagen deposition (19.5 ± 1.3% vs. 34.7 ± 2.1%, respectively, p < 0.0001, n = 10, Fig. 3B). Analysis of neo-angiogenesis in the infarcted area revealed no significant differences in the level of CD31-positive capillaries between the groups (37.2 ± 9.5 vessels/mm^2^ in MKEY-treated vs. 28.2 ± 7.8 vessels/mm^2^ in control mice, Fig. 3C). Moreover, we have previously shown that platelet-derived CCL5 and CXCL4 mediate monocyte and neutrophil recruitment. Here, CCL5 and CXCL4 release from platelets following TRAP-6 stimulation significantly increased neutrophil recruitment to immobilized platelets, and co-incubation with MKEY significantly reduced neutrophil recruitment (MKEY: 170.8 ± 12.0 vs. control 229.3 ± 6.3 neutrophils/mm^2^, n = 3–6, p < 0.05, Fig. 4A). We next analysed the recruitment of inflammatory cells in the infarcted area one day after myocardial I/R. Monocyte/macrophage infiltration was also reduced in the infarcted areas after MKEY treatment, as revealed by F4/80 staining, one day after I/R (107 ± 17.9 monocytes/mm^2^ vs. 203 ± 28.6 monocytes/mm^2^ in control group, n = 8, p < 0.05, Fig. 4B). Consistent with our *in vitro* observations, MKEY treatment significantly decreased post-ischemic neutrophil infiltration, as shown by specific esterase staining (MKEY: 144 ± 26.2 vs. control: 460 ± 128 neutrophils/mm^2^, n = 8, p < 0.05, Fig. 4B). However, the lymphocyte infiltration did not differ between the studied groups (Suppl. Fig. 2). Recent work has demonstrated involvement of NETs in the pathophysiology of myocardial infarction^27^. Incubation of neutrophils with LPS- or TRAP6-treated platelets, but not with LPS- or TRAP6 alone, led to a robust formation of NETs, which could be inhibited by the administration of MKEY (Suppl. Fig. 3), thereby confirming previous observations^23^. Interestingly, MKEY treatment almost completely abrogated the formation of neutrophil extracellular traps compared to control as reflected by the strongly reduced number of citrullinated histone 3 (H3cit) -positive cells in the infarcted tissue (MKEY: 21.7 ± 12.4 vs control 748.0 ± 72.0 cells/mm^2^, n = 3, p < 0.01, Fig. 4D).

## Discussion

In this study, we have demonstrated the efficiency of a synthetic peptide, MKEY, designed to specifically block CCL5-CXCL4 interactions, in the treatment of myocardial ischemia/reperfusion injury. Administration of MKEY significantly reduced infarction size due to a reduction of inflammation after MI. After treatment with MKEY, neutrophil and monocyte infiltration into the affected myocardial areas was reduced, which positively affected the infarction outcome and heart function. Previous studies have shown that circulating monocytes infiltrate the affected tissue in distinct waves after myocardial infarction, with classical Ly6C^hi^ CCR2^+^ monocytes being responsible for clearance of dead cells and the Ly6C^hi^ CCR2^−^ monocytes for tissue repair^4,28^. In addition, under healthy steady-state conditions the cardiac tissue is populated with resident macrophages. Recent studies have shown that tissue resident macrophages originate to a considerable part from embryonic yolk-sac-derived precursors, at least in the heart, liver and brain^29^. Under homeostatic conditions, the heart was found to be mainly populated by resident yolk-sac-derived macrophages Ly6C^low^ CCR2^−^ cells that are self-maintained by local proliferation^29^ and these can be replaced or replenished during cardiac inflammation by circulating Ly6C^hi^ CCR2^+^ cells. Although the roles of the chemokine receptors CCR2 and CCR5, as well as CX3CR1 on the influx of circulating monocytes into inflamed tissues has been well characterized^28,30^, the effects of the individual chemokines on resident macrophages is less well defined. The classical Ly6C^hi^ CCR2^+^ monocytes have been shown to use the CCL5 receptors CCR1 and −5 to enter the inflamed atherosclerotic aorta^31^. However, the role of CCL5 on the behaviour of resident macrophages is currently not known. Yet in a previous study implementing MKEY in a model of ischemic stroke, a strong inhibition of immigrated monocytes by MKEY could be observed, whereas the resident (yolk sac-derived) microglia were not affected by MKEY treatment^32^. In the present study, we also observed a decrease of cardiac monocyte content after treatment with MKEY. We have not been able to distinguish between resident and infiltrated cells, since the Ly6C^low^ CCR2^−^ monocytes accumulate in the infarcted area later after the acute hypoxic event (peaking after 7 days)^28^. Thus, in combination with the previous findings by Fan and colleagues^32^, we postulate that during the early phase after MI (after 1 day), MKEY mostly affects the inflammatory Ly6C^hi^ CCR2^+^ monocytes. Due to the particular distribution of the infarcted area through the heart, characteristic for ischemia/reperfusion injury, a significant decrease in infarction size after MKEY treatment allows the remote myocardium to compensate the lost tissue and thereby to preserve the regional contractility and heart function (Tables 1 and 2). The myocardium has a complex cellular structure, which besides cardiomyocytes includes fibroblasts and endothelial cells integrated in capillaries. During hypoxia, endothelial cells reorganize and start angiogenesis governed by angiogenic factors released directly after MI, e.g. VEGF or CXCL12^33^, while fibroblasts start to proliferate^34^. The cardiomyocytes however are undergoing apoptosis or even necrosis, as indicated by the findings in our study. However, unlike shown for blockade of CCL5^35^, angiogenesis was not affected, indicating that this side effect does not accompany MKEY treatment. In various interventional approaches, the diverse aspects of chemokine biology were addressed from different angles, particularly for CCL5, e.g. receptor-ligand interactions, prevention of the chemokine-glycosaminoglycan interaction, interfering with the receptor signalling or trafficking pathways, and prevention of heterodimer formation^36^, with varying success^37,38^.

Of all chemokines, the CC-chemokine receptors CCR1 and CCR5 and their ligand CCL5 appear to play a particular role in cardiovascular diseases. While low serum levels of CCL5 were associated with disease progression in atherosclerosis^39^, plasma levels of CCL5 were significantly elevated in patients with refractory ischemic symptoms versus stabilized patients^40^. By binding to its receptors, CCL5 mediates the arrest and transmigration of monocytes and T lymphocytes through the endothelium. However, it appears that CCL5 receptors exerted differential functions in induction and progression of cardiovascular diseases^41^. While CCR5 showed a protective role in atherosclerosis by a mechanism involving interleukin-10, CCR1 supported inflammation and neointima formation after vascular injury^42,43^. In contrast, deletion of CCR1 was shown to reduce tissue injury and preserve left ventricular function after myocardial infarction in mice^10^, whereas deletion of CCR5 enhanced inflammation, impaired recruitment of regulatory T cells and induced more severe cardiac dilation^44^. However, the administration of CCL5 antagonists or blocking antibodies could prevent both; the progression of atherosclerotic lesions by impairing the chemokine oligomerization^45,46^ and myocardial injury after infarction, by reducing leukocyte infiltration, chemokine expression and apoptosis in the affected myocardium^12,25^. Despite the evident beneficial effect of CCL5 antagonism in experimental studies, CCL5 blockade in clinical practice does not appear to be feasible, due to a potential compromise of the systemic immune response, delayed macrophage-mediated viral clearance and impaired normal T cell functions^47,48^. Local administration would be an alternative to prevent such side effects. For example, intramyocardial injection of CCL5 antagonists in combination with a protease-resistant form of the stem cell attractant CXCL12, embedded in biodegradable hydrogels led to reduction of tissue damage and to an improvement of cardiac function^26^. However, the clinical applicability of this approach needs to be established. Targeting the heteromerization of CCL5 with CXCL4 appears to be a realistic alternative approach to attenuate its functions without affecting the systemic immune response^38^. This notion is supported by the results in this study, which reveal that the inhibition of heteromer formation by MKEY reduced myocardial damage and neutrophil and monocyte infiltration into the affected tissue to a similar extent as full inhibition with CCL5 antagonists or blocking antibodies^12,25^.

Whereas the functions and the antagonism of CCL5 in myocardial infarction have been extensively studied, less is known about the role of CXCL4. CXCL4 is released from alpha-granules of activated platelets during platelet aggregation, and may modulate blood coagulation by binding to heparin-like molecules. Although first described as a chemoattractant for neutrophils and monocytes^49^, later studies have ascribed some of these CXCL4 activities to contaminations of other chemokines^50^. In fact, CXCL4 has been recognized as a physiological inhibitor of megakaryocytopoiesis and angiogenesis, rather than a classical leukocyte-recruiting chemokine^51^. Still, CXCL4 might promote inflammation, notably in cardiovascular disease. For example, genetic deletion of CXCL4 reduced atherosclerotic plaque and neointima formation in mice^52,53^. A recent study demonstrated that CXCL4 acts as a pro-inflammatory and mitogenic factor on smooth muscle cells, promoting remodelling after vascular injury^54^. During acute coronary syndrome, serum levels of CXCL4 increase in patients^55^, correlating with the severity of the myocardial injury^56^. On a mechanistic level, the enhancement of CCL5-function by CXCL4 appeared to be largely mediated by CCR1 requiring its third extracellular loop^57^, consistent with the role of CCR1 in myocardial ischemia/reperfusion injury^10^. Thus, CXCL4 appears to play an auxiliary role in CCL5-mediated CCR1 activation, a function that might be of crucial importance for the *in vivo* activity of CCL5. This notion is also supported by recent studies that implemented MKEY in mouse models of inflammatory disease. For example, MKEY was shown to suppresses abdominal aortic aneurysm formation and progression, reducing aortic diameter enlargement, preserving medial elastin fibres and smooth muscle cells, and attenuating mural macrophage infiltration, angiogenesis, and aortic metalloproteinase 2 and 9 expression^24^. It is known that cardiomyocytes express CCL5 and that the addition of CCL5 to cardiomyocytes significantly reduced myocyte contractility such as after MI, with no alterations in the Ca^2+^ transition^58^. Since the chemokines CCL5 and CXCL4 exert specific functions by heteromerization, blocking this by MKEY should not change the levels of these chemokines, yet it does block the function of the heteromers. Administration of MKEY reduced the infiltration of monocytes into ischemic brain tissue in a mouse model of stroke^32^. Nevertheless, it cannot be excluded that MKEY also has direct effects on cardiomyocytes. However, possible effects of CCL5 and CXCL4 on cardiomyocytes and their modulation by MKEY are subject to future investigation.

The contribution of platelets to reperfusion injury by promoting inflammatory reactions within the ischemic myocardium has been demonstrated in several earlier studies^59,60^. Particulary, platelet-neutrophil interactions provoke post-reperfusion cardiac dysfunction. MKEY treatment significantly decreased the adhesion of PMN to immobilized TRAP-6 stimulated platelets under flow conditions. Moreover, MKEY interfered with platelet-neutrophil interactions in an aseptic and a septic mouse model of acute lung injury, diminishing neutrophil lung extravasation, oedema formation and neutrophil elastase release^21^. In addition, the administration of MKEY decreased the severity of ventilator-induced lung injury in mice^23^. Interestingly, CCL5 and CXCL4 were found to induce NET release by neutrophils and MKEY could effectively prevent NET formation when neutrophils were pre-incubated with platelets. These findings were corroborated by our observations showing that NET-release induced by TRAP-6- or LPS-activated platelets could be attenuated by the addition of MKEY. In a previous study implementing a mouse model of myocardial I/R, the administration of the NET-degrading enzyme DNAseI led to a reduction of infarct size and to an improvement of ejection fraction^27^, indicating that ischemic myocardial I/R damage is driven by NET formation. When myocardial I/R was performed in mice deficient for peptidylarginine deiminase 4 (PAD4), having neutrophils defective in NET-formation, infarct size was significantly reduced^27^.

Although our study takes a different approach, blocking CCL5/CXCL4 interactions by a peptide inhibitor, we could also link the observed reduction in myocardial I/R damage after administration of MKEY with a reduction of neutrophil infiltration and an associated decrease in NET formation.

A limitation of this study is the administration of the MKEY peptide before the experimental induction of myocardial I/R, thereby affecting clinical translation of our findings. Since this study was primarily conceived to investigate mechanistic aspects of the CCL5/CXCL4 complex in myocardial I/R, we have opted to pre-treat the animals with the inhibitory compound before coronary occlusion to achieve plasma levels of the compounds at the moment of treatment. Due to this pre-administration of the compounds, the observed reduction in infarct sizes might be due to an attenuation of inflammation before the induction of ischemia, resulting in less post-infarction damage and scar formation. Nevertheless, results from our model experiments and from previous work of others have demonstrated direct effects of the MKEY peptide on leukocyte recruitment and behaviour, both affecting the inflammatory response after ischemia. Thus, we can conclude that disruption of the CCL5/CXCL4 complex does attenuate the post-ischemic inflammatory reaction. Although infusion of the compound before the induction of ischemia might hamper clinical applicability, the data still demonstrate that our approach can in principle present a useful strategy for reducing tissue damage.

In conclusion, the peptide MKEY might represent a new therapeutic approach for the treatment of atherosclerosis and myocardial infarction. This strategy could have potential clinical impact, having advantage over direct antagonism of chemokines or their receptors, by preserving the therapeutic effect of specifically blocking chemokines, yet also reducing the side effects and maintaining normal immune defence.

## Material and Methods

### Ethics

Studies on human subjects involved blood sampling for platelet and neutrophil isolation and were performed conform the declaration of Helsinki and approval was granted by the local institutional ethics boards (Ethikkommission bei der Medizinischen Fakultät der LMU München, and the medisch-ethische toetsingscommissie (METC) at Maastricht University). All animal experiments and study protocols were performed at the University Hospital of the RWTH Aachen and approved by local authorities, complying with German animal protection laws and performed according to the guidelines from Directive 2010/63/EU of the European Parliament (ethics approval granted by Landesamt für Natur- und Verbaucherschutz (LANUV) Nordrhein-Westfalen (NRW) Nrs. 50.203.2-AC 37, 26/05 and 84-02.04.2013.A185).

### Animal Treatment

C57BL/6 mice (11–12 per group) were treated with MKEY or scrambled control MKEY (sMKEY) (Suppl. Fig. 1), synthesized by t-Boc-based solid-phase peptide synthesis as described^20^. For infarction size and functional parameter analysis, MKEY or sMKEY was dissolved at 2.27 mg/ml (1 mmol/l) in 0.01% formic acid and loaded in 100 µl Alzet type 1007D osmotic pumps (0.5 µl/hour, Charles River, Cologne, Germany), resulting in a dose of 1.3 mg/kg i.v per 24 hours. One day before I/R, the mice were anesthetized by ketamine and xylazine (100 and 10 mg/kg i.p., respectively) and the pumps were implanted for intravenous delivery into the jugular vein, according to manufacturer’s instructions. One week after I/R, the mice underwent echocardiography and intraventricular pressure measurements. Finally, one day or one week after I/R, the mice were anesthetized with an intraperitoneal injection of ketamine and xylazine (100 mg/kg and 10 mg/kg, respectively) and hearts were excised, fixed in formalin and embedded in paraffin for further analysis.

To avoid supplementary burden of animals according to the new European animal experimental regulations, mice undergoing analysis after short period of time (24 hours) were treated one day before and on day of I/R intraperitoneally with 9 mg/kg MKEY and 9 mg/kg sMKEY as described^20,21^ (Suppl. Fig. 1).

### Myocardial ischemia and reperfusion

11–12 week-old male mice were intubated under general anesthesia (100 mg/kg ketamine, 10 mg/kg xylazine, i.p.) and positive-pressure ventilated with oxygen and 0.2% isoflurane using a rodent respirator^61^. Hearts were exposed by left thoracotomy and MI was produced by suture occlusion of the left anterior descending artery (LAD) over a silicone tube. After 60 min of ischemia, the tube and suture were removed to permit reperfusion. Sham-operated mice (10 per group) underwent all surgical actions except LAD occlusion. The muscle layer and skin incision were closed with a silk suture. Either one day or one week after I/R, the hearts were harvested for further analysis. The mice received buprenorphine (0.1 mg/kg) as analgesia before and for up to 5 days after surgery. Surgery was accompanied by moderate lethality in mice, with approximately 20% of the animals dying within 24 hours, no differences were observed between the treated groups.

### Echocardiography

Two-dimensional and M-mode echocardiographic measurements were performed on a small-animal ultrasound imager (Vevo 770, FUJIFILM Visualsonics, Toronto, Canada). Both procedures were performed before and after I/R. Mice were anesthetized by mask with 1.5% isoflurane and placed in supine position on a warming pad. The ejection fraction, cardiac output, left ventricular end-diastolic (LVEDD), left ventricular end-systolic (LVESD) diameters, heart rate and heart weight were recorded and analysed^26^.

### *In vivo* assessment of cardiac function

One week after I/R (Suppl. Fig. 1), the mice were anesthetized with ketamine/xylazine as above and a Millar Mikro-Tip^®^ Pressure Transducer Catheter (Millar Instruments, Houston, Tx) was introduced into the right common carotid artery. After ligation, the catheter was slowly advanced into the left ventricle, and blood pressure in the left ventricle, as well as increase (dP/dtmax) and decrease (dP/dtmin) in left ventricular pressure were measured.

### Evans-blue/tetrazolium staining

AAR and infarction size were also measured one day after reperfusion (Suppl. Fig. 1). The hearts were excised from mice treated with MKEY and sMKEY (8 per group) and washed with PBS, the ligature over the LAD was renewed and 200 µl Evans-blue was perfused through the aorta. After freezing at −20 °C for one hour, the hearts were cut in 5 slices, and incubated in tetrazolium solution at 37 °C for 10 min, followed by 10% formaldehyde for 10 min. The slices were fixed between microscopic slides for photography and measurements. The total ventricle area, the blue-stained normally perfused area, red-stained injured but still viable area and white infarcted area were measured using ImageJ. AAR was calculated as the difference between the total and blue-stained area and express as percent from total ventricle area. Infarction size was calculated as white unstained area and expressed as percent of AAR^61^. Of the initial 8 animals per group, 6 were taken into the analysis (1 of each group died after surgery, 1 sample per group was lost during pre-analytical processing).

### Histology and immunohistochemistry

To evaluate MI size, serial sections (10–12 sections per mouse, 400 µm apart, up to the mitral valve) were stained with Gomori’s 1-step trichrome stain. The infarcted area was determined for all sections using Diskus software (Hilgers, Königswinter, Germany) and expressed as a percentage of total left ventricular volume. Blue-stained collagen content was analyzed with Cell P Software (Olympus, Hamburg, Germany) and expressed as a percentage of the infarcted area. Serial sections (3 sections per mouse, 400 µm apart) were stained to analyse the infarcted area for neutrophil content (specific esterase, Sigma, St. Louis, MO), neutrophil extracellular trap formation (H3cit, abcam 5103, Cambridge, UK), macrophage content (F4/80), lymphocytes (CD3, both Serotec, Oxford, UK) apoptotic cells (TUNEL, MEBSTAIN apoptosis kit II, MBL, Woburn, MA, USA) and CD31-positive capillaries (Santa Cruz, Santa Cruz, CA). Positive-stained cells or vessels were counted in six different fields per section and expressed as cells or vessels per mm^2^.

#### *In vitro* NET formation

Human neutrophils were isolated from the blood of healthy donors, after obtaining informed consent as described previously^62,63^. Isolated neutrophils were resuspended at (4 × 10^5^) in RPMI 1640 medium with 1% fetal calf serum. Citrate-anticoagulated whole blood was obtained from healthy donors and platelets were isolated as previously described^64^. Neutrophils (2 × 10^5^) were seeded on poly-L-lysine coated glass coverslips and incubated with platelets (1 × 10^7^) pretreated with lipopolysaccharide (LPS; 5 µg/mL) or thrombin receptor activator peptide (TRAP; 50 µM) with or without MKEY (10 µM) for 30 minutes at 37 °C. After 30 minutes of further incubation, samples were fixed with 2% PFA for 10 minutes at 37 °C, washed with PBS and incubated with Syto13^®^ green fluorescent nucleic acid stain (1 µM) for 20 minutes. Images were obtained with a fluorescence microscope (DM2000; Leica, Wetzlar, Germany).

#### Neutrophil adhesion under flow

Platelets were isolated from citrate-anticoagulated whole blood of healthy volunteers as described previously^64^ and resuspended at a concentration of 2 × 10^7^/mL. Washed platelets were immobilized on soluble rat-tail collagen-treated (30 µg/mL) glass cover slips (Menzel, 24 × 60 mm, #1.5) for 1.5 hours at 37 °C in a moisture chamber. Nonspecific binding was blocked with 0.5% human serum albumin (HSA) in HEPES buffer for 30 min at 37 °C. A confluent layer of spread platelets was formed, which was examined by phase contrast microscopy (EVOS-FL) before and after flow perfusion and was not affected by flow shear stress. Human neutrophils were isolated from the blood of healthy donors, after obtaining informed consent as described above. Isolated neutrophils were resuspended at (1 × 10^6^) in HBSS with 10 mM HEPES (pH 7.4) and 0.2% HSA (HHHSA buffer) and labeled with 1 µM Syto-13 (Thermofisher Scientific, Waltham, MA). Platelets were activated with 50 µM TRAP-6 in presence or absence of MKEY or sMKEY (1 µg/mL) for 1 h at 37 °C. Platelet-coated glass cover slips were assembled in parallel flow chambers (0.4 mm Luer sticky slides, ibidi®, Martinsried, Germany) and then mounted in a fluorescent microscope (EVOS-FL) using a 10x objective and a CCD camera. Prior to neutrophil perfusion, 2 mM MgCl_2_ and 3 mM CaCl_2_ was added into the cell suspensions and perfused for 2 min at a shear stress of 1 dynes/cm^2^. Following 2 min of perfusion, the number of firmly adherent cells per mm^2^ (no movement for >10 s) was counted in 6 different fields within 5 min.

#### Statistical analysis

Data represent mean ± SEM. Statistical analysis was performed with Prism 4 software (GraphPad). Means of two groups were compared with unpaired Student-t test and of more than two groups with 1-way ANOVA followed by Newman-Keuls post-hoc-test, as indicated. P-values of < 0.05 were considered significant.

## Electronic supplementary material

Supplementary Dataset 1

Supplementary video 1 - Sham

Supplementary video 2 - Control

Supplementary video 3 - MKEY

## References

[1] Tourki B, Halade G (2017). Leukocyte diversity in resolving and nonresolving mechanisms of cardiac remodeling. FASEB J.

[2] Horckmans M (2017). Neutrophils orchestrate post-myocardial infarction healing by polarizing macrophages towards a reparative phenotype. Eur Heart J.

[3] Liehn EA, Postea O, Curaj A, Marx N (2011). Repair after myocardial infarction, between fantasy and reality: the role of chemokines. J Am Coll Cardiol.

[4] Swirski FK, Nahrendorf M (2013). Leukocyte behavior in atherosclerosis, myocardial infarction, and heart failure. Science.

[5] Frangogiannis NG (2015). Interleukin-1 in cardiac injury, repair, and remodeling: pathophysiologic and translational concepts. Discoveries.

[6] Ferdinandy P, Hausenloy DJ, Heusch G, Baxter GF, Schulz R (2014). Interaction of risk factors, comorbidities, and comedications with ischemia/reperfusion injury and cardioprotection by preconditioning, postconditioning, and remote conditioning. Pharmacol Rev.

[7] Lecour S (2014). ESC working group cellular biology of the heart: position paper: improving the preclinical assessment of novel cardioprotective therapies. Cardiovasc Res.

[8] Koenen RR, Weber C (2011). Chemokines: established and novel targets in atherosclerosis. EMBO Mol Med.

[9] Dewald O (2005). CCL2/Monocyte Chemoattractant Protein-1 regulates inflammatory responses critical to healing myocardial infarcts. Circulation Research.

[10] Liehn EA (2008). Ccr1 deficiency reduces inflammatory remodelling and preserves left ventricular function after myocardial infarction. J Cell Mol Med.

[11] Liehn EA (2010). A new monocyte chemotactic protein-1/chemokine CC motif ligand-2 competitor limiting neointima formation and myocardial ischemia/reperfusion injury in mice. J Am Coll Cardiol.

[12] Montecucco F (2012). CC chemokine CCL5 plays a central role impacting infarct size and post-infarction heart failure in mice. Eur Heart J.

[13] Alard JE (2015). Recruitment of classical monocytes can be inhibited by disturbing heteromers of neutrophil HNP1 and platelet CCL5. Sci Transl Med.

[14] Sorensen LN, Paludan SR (2004). Blocking CC chemokine receptor (CCR) 1 and CCR5 during herpes simplex virus type 2 infection *in vivo* impairs host defence and perturbs the cytokine response. Scand J Immunol.

[15] Glass WG (2006). CCR5 deficiency increases risk of symptomatic West Nile virus infection. J Exp Med.

[16] Serbina NV, Pamer EG (2006). Monocyte emigration from bone marrow during bacterial infection requires signals mediated by chemokine receptor CCR2. Nat Immunol.

[17] Anders HJ (2003). CC chemokine ligand 5/RANTES chemokine antagonists aggravate glomerulonephritis despite reduction of glomerular leukocyte infiltration. J Immunol.

[18] Petersen F, Bock L, Flad HD, Brandt E (1999). Platelet factor 4-induced neutrophil-endothelial cell interaction: involvement of mechanisms and functional consequences different from those elicited by interleukin-8. Blood.

[19] von Hundelshausen P (2005). Heterophilic interactions of platelet factor 4 and RANTES promote monocyte arrest on endothelium. Blood.

[20] Koenen RR (2009). Disrupting functional interactions between platelet chemokines inhibits atherosclerosis in hyperlipidemic mice. Nat Med.

[21] Grommes J (2012). Disruption of platelet-derived chemokine heteromers prevents neutrophil extravasation in acute lung injury. Am J Respir Crit Care Med.

[22] von Hundelshausen, P. *et al*. Chemokine interactome mapping enables tailored intervention in acute and chronic inflammation. *Sci Transl Med* **9**, 10.1126/scitranslmed.aah6650 (2017). 10.1126/scitranslmed.aah6650 28381538

[23] Rossaint J (2014). Synchronized integrin engagement and chemokine activation is crucial in neutrophil extracellular trap-mediated sterile inflammation. Blood.

[24] Iida Y (2013). Peptide inhibitor of CXCL4-CCL5 heterodimer formation, MKEY, inhibits experimental aortic aneurysm initiation and progression. Arterioscler Thromb Vasc Biol.

[25] Braunersreuther V (2010). Chemokine CCL5/RANTES inhibition reduces myocardial reperfusion injury in atherosclerotic mice. Journal of Molecular and Cellular Cardiology.

[26] Projahn D (2014). Controlled intramyocardial release of engineered chemokines by biodegradable hydrogels as a treatment approach of myocardial infarction. J Cell Mol Med.

[27] Savchenko AS (2014). VWF-mediated leukocyte recruitment with chromatin decondensation by PAD4 increases myocardial ischemia/reperfusion injury in mice. Blood.

[28] Nahrendorf M (2007). The healing myocardium sequentially mobilizes two monocyte subsets with divergent and complementary functions. J Exp Med.

[29] Epelman S (2014). Embryonic and adult-derived resident cardiac macrophages are maintained through distinct mechanisms at steady state and during inflammation. Immunity.

[30] Tacke F (2007). Monocyte subsets differentially employ CCR2, CCR5, and CX3CR1 to accumulate within atherosclerotic plaques. J Clin Invest.

[31] Soehnlein O (2013). Distinct functions of chemokine receptor axes in the atherogenic mobilization and recruitment of classical monocytes. EMBO Mol Med.

[32] Fan, Y. *et al*. MKEY, a Peptide Inhibitor of CXCL4-CCL5 Heterodimer Formation, Protects Against Stroke in Mice. *J Am Heart Assoc* **5**, 10.1161/JAHA.116.003615 (2016). 10.1161/JAHA.116.003615 PMC5079025 27633389

[33] Kanzler I (2013). Differential roles of angiogenic chemokines in endothelial progenitor cell-induced angiogenesis. Basic Res Cardiol.

[34] Ma Y, Iyer RP, Jung M, Czubryt MP, Lindsey ML (2017). Cardiac Fibroblast Activation Post-Myocardial Infarction: Current Knowledge Gaps. Trends Pharmacol Sci.

[35] Westerweel PE, Rabelink TJ, Rookmaaker MB, Grone HJ, Verhaar MC (2008). RANTES is required for ischaemia-induced angiogenesis, which may hamper RANTES-targeted anti-atherosclerotic therapy. Thromb Haemost.

[36] Proudfoot AE, Power CA, Rommel C, Wells TN (2003). Strategies for chemokine antagonists as therapeutics. Semin Immunol.

[37] Horuk R (2009). Chemokine receptor antagonists: overcoming developmental hurdles. Nat Rev Drug Discov.

[38] Koenen RR, Weber C (2010). Therapeutic targeting of chemokine interactions in atherosclerosis. Nat Rev Drug Discov.

[39] Cavusoglu E (2007). Low plasma RANTES levels are an independent predictor of cardiac mortality in patients referred for coronary angiography. Arterioscler Thromb Vasc Biol.

[40] Kraaijeveld AO (2007). CC chemokine ligand-5 (CCL5/RANTES) and CC chemokine ligand-18 (CCL18/PARC) are specific markers of refractory unstable angina pectoris and are transiently raised during severe ischemic symptoms. Circulation.

[41] Kanzler I, Liehn EA, Koenen RR, Weber C (2012). Anti-inflammatory therapeutic approaches to reduce acute atherosclerotic complications. Curr Pharm Biotechnol.

[42] Braunersreuther V (2007). Ccr5 but not Ccr1 deficiency reduces development of diet-induced atherosclerosis in mice. Arterioscler Thromb Vasc Biol.

[43] Zernecke A (2006). Deficiency in CCR5 but not CCR1 protects against neointima formation in atherosclerosis-prone mice: involvement of IL-10. Blood.

[44] Dobaczewski M, Xia Y, Bujak M, Gonzalez-Quesada C, Frangogiannis NG (2010). CCR5 signaling suppresses inflammation and reduces adverse remodeling of the infarcted heart, mediating recruitment of regulatory T cells. Am J Pathol.

[45] Braunersreuther V (2008). A Novel RANTES Antagonist Prevents Progression of Established Atherosclerotic Lesions in Mice. Arterioscler Thromb Vasc Biol.

[46] Veillard NR (2004). Antagonism of RANTES receptors reduces atherosclerotic plaque formation in mice. Circ Res.

[47] Makino Y (2002). Impaired T cell function in RANTES-deficient mice. Clin Immunol.

[48] Tyner JW (2005). CCL5-CCR5 interaction provides antiapoptotic signals for macrophage survival during viral infection. Nat Med.

[49] Deuel TF (1981). Platelet factor 4 is chemotactic for neutrophils and monocytes. Proc Natl Acad Sci USA.

[50] Petersen F (2000). Is platelet factor-4 a chemokine?. Eur Cytokine Netw.

[51] Karshovska E, Weber C, von Hundelshausen P (2013). Platelet chemokines in health and disease. Thromb Haemost.

[52] Sachais BS (2007). Elimination of platelet factor 4 (PF4) from platelets reduces atherosclerosis in C57Bl/6 and apoE−/− mice. Thromb Haemost.

[53] Wang H (2009). Core2 1-6-N-glucosaminyltransferase-I deficiency protects injured arteries from neointima formation in ApoE-deficient mice. Arterioscler Thromb Vasc Biol.

[54] Shi G (2013). Platelet factor 4 mediates vascular smooth muscle cell injury responses. Blood.

[55] Blanchet X (2014). Inflammatory role and prognostic value of platelet chemokines in acute coronary syndrome. Thromb Haemost.

[56] Caimi G (2005). Plasma markers of platelet and polymorphonuclear leukocyte activation in young adults with acute myocardial infarction. Clin Hemorheol Microcirc.

[57] Kramp BK (2013). Exchange of extracellular domains of CCR1 and CCR5 reveals confined functions in CCL5-mediated cell recruitment. Thromb Haemost.

[58] Kelly KM (2014). CCR5 inhibition prevents cardiac dysfunction in the SIV/macaque model of HIV. J Am Heart Assoc.

[59] Lefer AM, Campbell B, Scalia R, Lefer DJ (1998). Synergism between platelets and neutrophils in provoking cardiac dysfunction after ischemia and reperfusion: role of selectins. Circulation.

[60] Kupatt C (2000). c7E3Fab reduces postischemic leukocyte-thrombocyte interaction mediated by fibrinogen. Implications for myocardial reperfusion injury. Arterioscler Thromb Vasc Biol.

[61] Curaj, A., Simsekyilmaz, S., Staudt, M. & Liehn, E. Minimal invasive surgical procedure of inducing myocardial infarction in mice. *J. Vis. Exp*., e52197, 10.3791/52197 (2015). 10.3791/52197 PMC4542456 25992740

[62] Brinkmann, V., Laube, B., Abu Abed, U., Goosmann, C. & Zychlinsky, A. Neutrophil extracellular traps: how to generate and visualize them. *J. Vis. Exp*., 10.3791/1724 (2010). 10.3791/1724 PMC3125121 20182410

[63] Saffarzadeh M (2014). Characterization of rapid neutrophil extracellular trap formation and its cooperation with phagocytosis in human neutrophils. Discoveries.

[64] Feijge MA (1998). Inter-individual variability in Ca^2+^ signalling in platelets from healthy volunteers: effects of aspirin and relationship with expression of endomembrane Ca^2+^ -ATPases. Br J Haematol.

