# Supplementary material for: Blocking CCL5-CXCL4 heteromerization preserves heart function after myocardial infarction by attenuating leukocyte recruitment and NETosis
Source: Sci Rep. 2018 Jul 13;8:10647. doi: 10.1038/s41598-018-29026-0 (PMC6045661; doi:10.1038/s41598-018-29026-0)
Supplement: Supplementary file 1 — Supplementary Dataset 1 [file 41598_2018_29026_MOESM1_ESM.pdf]

Supplementary information

Blocking CCL5-CXCL4 heteromerization preserves heart function after myocardial infarction by attenuating leukocyte recruitment and NETosis

Running Title: Targeting chemokine heteromers in heart infarction

Tanja Vajen[1,§], PhD; Rory R. Koenen[1,4,§,#], PhD; Isabella Werner[2,§], PhD; Mareike Staudt[2], MS; Delia Projahn[2,5], PhD; Adelina Curaj[2,5,6], PhD; Tolga Taha Sönmez[2,7,8], MD; Sakine Simsekyilmaz, PhD[2], David Schumacher[2], BS; Julia Möllmann[2,10], MS; Tilman M. Hackeng, PhD[1]; Philipp von Hundelshausen[4,9], MD; Christian Weber[1,4,9,*], MD; Elisa A. Liehn[2,10,11*], MD, PhD

[1]Cardiovascular Research Institute Maastricht (CARIM), Department of Biochemistry, Maastricht University, Maastricht, the Netherlands. [2]Institute for Molecular Cardiovascular Research (IMCAR), RWTH Aachen University, Germany. [4]Institute for Cardiovascular Prevention (IPEK), LMU Munich, Munich, Germany. [5]Department of Experimental Molecular Imaging, RWTH Aachen University, Germany. [6]Victor Babes National Institute of Pathology, Bucharest, Romania. [7]Department of Oral and Maxillofacial Surgery, Karlsruhe City Hospital of Freiburg University, Germany. [8]Department of Oral and Maxillofacial Surgery, University Medical Center Hamburg-Eppendorf, Germany. [9]DZHK (German Centre for Cardiovascular Research), partner site Munich Heart Alliance, Munich, Germany, [10]Department of Cardiology, Pulmonology, Angiology and Intensive Care, University Hospital Aachen, Germany, [11]Human Genetic Laboratory, University of Medicine and Pharmacy, Craiova, Romania

Supplementary Figure 1

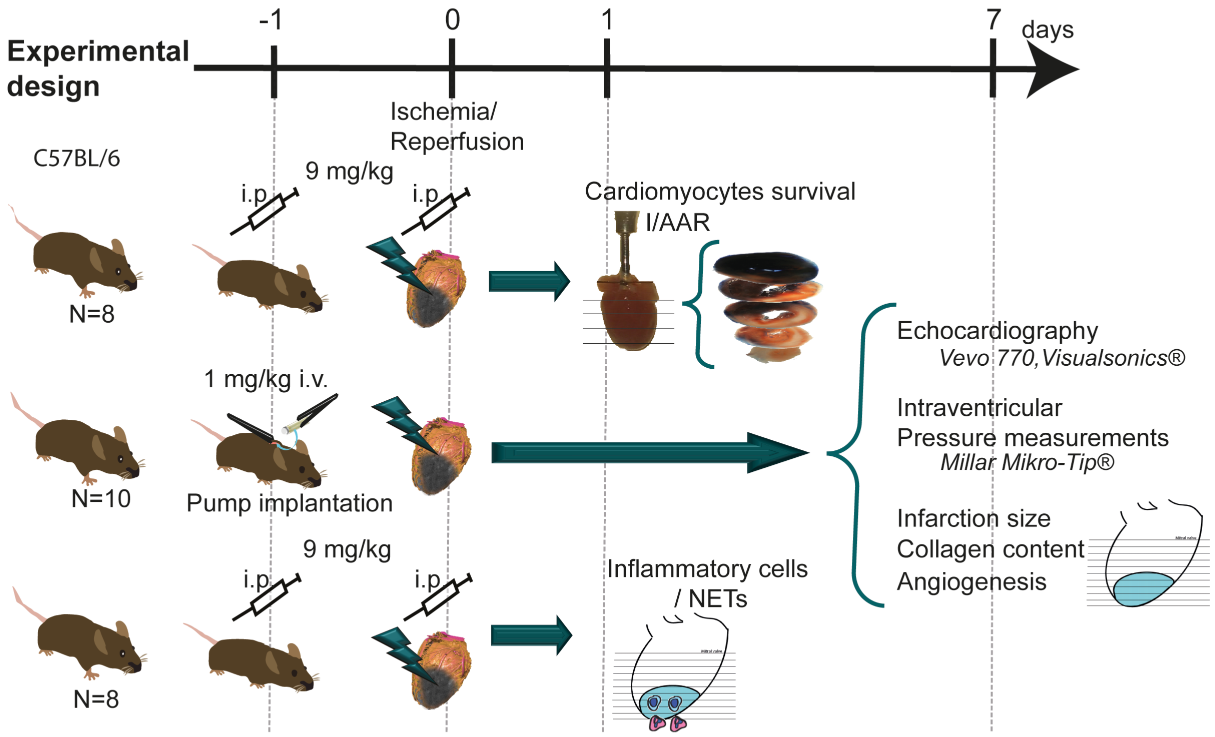

Figure S1. *Experimental study design.*

Time course of experiment in days. MKEY or control peptides were administrated intraperitoneally (i.p.) at 9 mg/kg or intravenously by osmotic minipumps at 1 mg/kg before induction of I/R.

Supplementary Figure 2

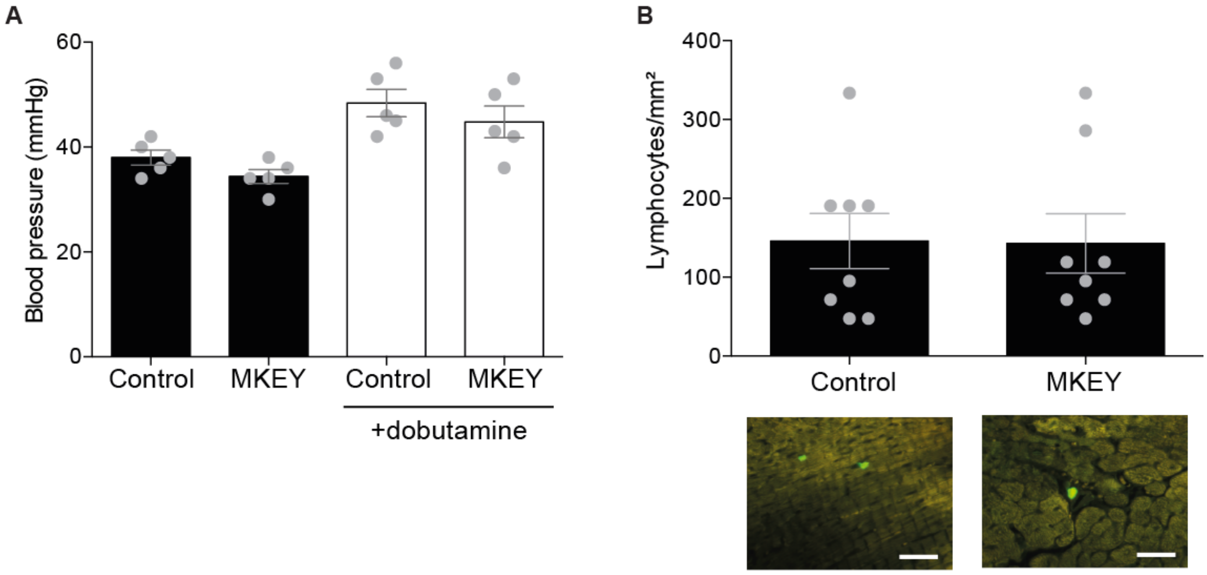

Figure S2. *Effects of MKEY treatment on blood pressure and infarct lymphocyte content*

(A) Blood pressures were assessed using intraventricular Millar catheter after control or MKEY treatment, without or with dobutamine stimulation (n=5). (B) Lymphocyte infiltration (CD3 staining) in MKEY- and sMKEY-treated mice, one day after I/R (n=8); Scale bar 50 µm.

Supplementary Figure 3

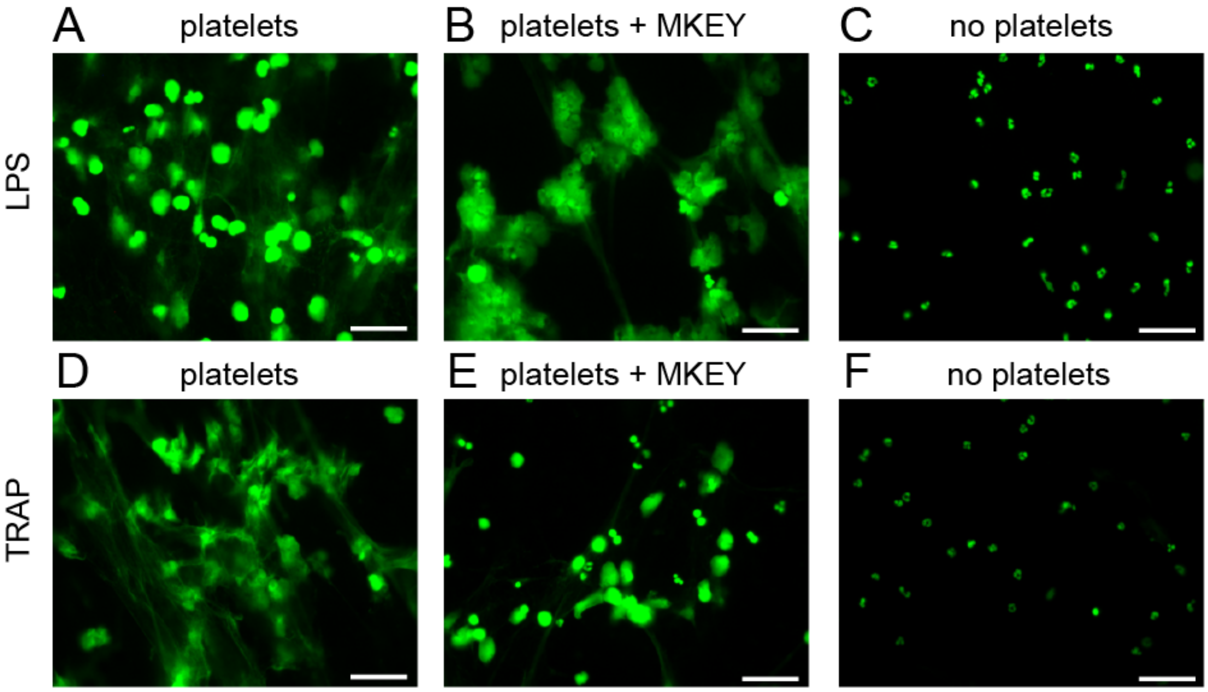

Figure S3. *Inhibition of NETosis by MKEY.*

Isolated washed human platelets were treated with LPS (5µg/mL, A,B) or TRAP (50 µM, D,E) for 30 minutes prior to addition to neutrophils. After 30 minutes of incubation without or with MKEY (10µM), the neutrophils were fixed and stained with Syto13 to visualize DNA. Treatment of neutrophils with LPS or TRAP in the absence of platelets did not result in NETosis (C,F). Scale bar 100 µm.
